# Development of a predictive nomogram for intermediate-risk differentiated thyroid cancer patients after fixed 3.7GBq (100mCi) radioiodine remnant ablation

**DOI:** 10.3389/fendo.2024.1361683

**Published:** 2024-05-30

**Authors:** Lu Lu, Qiang Li, Zhao Ge, Yanqi Lu, Chunhao Lin, Jinfu Lv, Jinquan Huang, Xingyu Mu, Wei Fu

**Affiliations:** Department of Nuclear Medicine, Guilin Medical University Affiliated Hospital, Guilin, China

**Keywords:** intermediate-risk, differentiated thyroid cancer, radioiodine remnant ablation, predictive nomogram, thyroglobulin, cervical lymph node metastasis

## Abstract

**Objectives:**

The objective of this study was to develop a predictive nomogram for intermediate-risk differentiated thyroid cancer (DTC) patients after fixed 3.7GBq (100mCi) radioiodine remnant ablation (RRA).

**Methods:**

Data from 265 patients who underwent total thyroidectomy with central lymph node dissection (CND) and received RRA treatment at a single institution between January 2018 and March 2023 were analyzed. Patients with certain exclusion criteria were excluded. Univariate and multivariate logistic regression analyses were performed to identify risk factors for a non-excellent response (non-ER) to RRA. A nomogram was developed based on the risk factors, and its performance was validated using the Bootstrap method with 1,000 resamplings. A web-based dynamic calculator was developed for convenient application of the nomogram.

**Results:**

The study included 265 patients with intermediate-risk DTC. Significant differences were found between the ER group and the non-ER group in terms of CLNM>5, Hashimoto’s thyroiditis, sTg level, TgAb level (P < 0.05). CLNM>5 and sTg level were identified as independent risk factors for non-ER in multivariate analysis. The nomogram showed high accuracy, with an area under the curve (AUC) of 0.833 (95% CI = 0.770–0.895). The nomogram’s predicted probabilities aligned closely with actual clinical outcomes.

**Conclusions:**

This study developed a predictive nomogram for intermediate-risk DTC patients after fixed 3.7GBq (100mCi) RRA. The nomogram incorporates CLNM>5 and sTg levels as risk factors for a non-ER response to RRA. The nomogram and web-based calculator can assist in treatment decision-making and improve the precision of prognosis information. Further research and validation are needed.

## 1 Introduction

Radioactive iodine therapy (RAIT) is a pivotal treatment for differentiated thyroid carcinoma (DTC) following total thyroidectomy, categorized into three modalities based on therapeutic objectives: 1) Radioiodine remnant ablation (RRA) aims to eliminate residual thyroid tissue post-surgery; 2) Radioiodine adjuvant therapy (RAT) targets undetected metastatic or residual lesions; 3) Radioiodine treatment (RT) addresses inoperable local or distant DTC metastases (1, 2). RRA is instrumental in post-thyroidectomy DTC management, enhancing serum thyroglobulin (Tg) level stratification, patient monitoring, the sensitivity of ^131^I whole-body scans for metastasis detection, and postoperative restaging (1, 3, 4). RAT extends beyond eliminating residual tissue to addressing latent lesions, thereby augmenting disease-free survival (DFS) rates (5, 6). Clinicians must grasp Tg level stratification in postoperative DTC patients and evaluate recurrence risk, especially in intermediate or low-risk cases, to optimize remnant ablation strategies.

The recommended ^131^I dose for RRA varies from 1.11 to 3.70 GBq (30 to 100 mCi) (1). Research shows comparable efficacy between 1.11 and 3.70 GBq doses in low or intermediate-risk DTC patients post-surgery, with lower doses minimizing short-term adverse effects. However, higher doses are suggested for patients with substantial thyroid remnants or as adjuvant treatment. Dose determination should integrate clinical and pathological characteristics, mortality and recurrence risks, and dynamic assessment, favoring tailored approaches over standardized low (1.11 GBq) or high (3.70 GBq) doses (7). Factors influencing RRA dose escalation include larger residual thyroid tissue, elevated thyroglobulin levels, and additional risk indicators. In scenarios where patients exhibit stimulated thyroglobulin (sTg) levels above 10ng/ml and are classified as high-risk for recurrence, RAT with doses exceeding 3.70 GBq is advisable (8, 9).

Studies associate factors like tumor size, elevated serum sTg levels, lymph node capsular invasion, N1a classification, and distant metastases with increased RRA failure risk (10). A serum sTg level below 2ng/mL is a vital predictor for positive response, with higher pre-ablation sTg levels indicating a potential initial treatment inadequacy (11). Hence, sTg levels serve as a predictive measure for RRA success. Notably, prior research involving patients with sTg levels above 10, classified as high-risk, and exhibiting structural lesions, employed varying ^131^I doses, potentially introducing biases. Therefore, a distinct risk predictive model to predict the response to fixed 3.7GBq (100mCi) RRA with intermediate-risk DTC is necessary.

The predominant staging systems for DTC are the TNM system and recurrence risk stratification. DTC typically demonstrates low disease-specific mortality. These staging systems primarily predict recurrence risk in patients rather than the efficacy of initial RRA. In contrast, nomograms, which have been developed for most cancer types, often outperform traditional staging methods (12–14). Consequently, many consider nomograms a potential alternative or new standard (15). This study aims to establish a risk nomogram utilizing clinicopathologic data from 265 intermediate-risk DTC patients who underwent a fixed dose of 3.7GBq (100mCi) RRA.

## 2 Patients and methods

### 2.1 Patients and study design

This retrospective study focused on patients who underwent thyroidectomy and RRA from January 2018 to March 2023 at the Affiliated Hospital of Guilin Medical University. Eligible participants had undergone thyroidectomy and central lymph node dissection (CND), with histopathologically confirmed DTC. They were categorized into the intermediate-risk group for recurrence and received an fixed RRA dose of 3.7GBq (100mCi). The intermediate risk group was defined in accordance with the ATA risk criteria (1). This group includes patients with the following characteristics: (1) microscopic invasion of the tumor into the perithyroidal soft tissues, (2) the presence of radioactive iodine (RAI)-avid metastatic foci in the neck as observed on the first post-treatment whole-body RAI scan, (3) aggressive histology (e.g., tall cell, hobnail variant, columnar cell carcinoma), (4) papillary thyroid cancer with vascular invasion, (5) clinical N1 or >5 pathologic N1 with all involved lymph nodes <3 cm in the largest dimension, and (6) multifocal papillary microcarcinoma with extrathyroidal extension (ETE) and BRAFV600E mutation (if known). The exclusion criteria included: sTg levels exceeding 10 ng/ml; serum thyroglobulin antibody (TgAb) positivity (>115 U/mL); evidence of cervical lymph node metastasis (CLNM) or distant metastasis (DM) post RRA whole-body scintigraphy (Rx-WBS); being under 18 years of age; and insufficient clinicopathologic data for analysis.

### 2.2 RRA and follow-up

Patients underwent RRA following surgery (total thyroidectomy with central lymph node dissection [CND]). Indications for total thyroidectomy included: (a) primary tumor size exceeding 4 cm; (b) bilateral/multiple lesions; (c) extrathyroidal extension; (d) clinical evidence of lymph node or distant metastasis. Ipsilateral CND was performed for all patients, while bilateral CND was conducted in cases of bilateral carcinoma or clinically suspicious nodal disease in the contralateral central compartment. Lateral neck dissection (LND), encompassing levels II–V, was reserved for clinically or pathologically confirmed metastatic lateral neck lymph nodes.

Prior to RRA, patients adhered to a low-iodine diet for 14 days, guided by our nutrition experts, avoiding iodine-containing medications. RRA was administered post levothyroxine (LT4) withdrawal, aligned with the American Joint Committee on Cancer (AJCC) TNM staging and recurrence risk stratification, at a dose of 3.7GBq (100mCi). Pre-RRA routine biochemical (serum thyrotropin [TSH], sTg, anti-Tg antibody [TgAb]) and imaging (ultrasonography, computed tomography) examinations were conducted. Post-RRA, LT4 was prescribed for TSH suppression therapy, and Rx-WBS was performed 2–3 days later.

Follow-up visits post-RRA included TSH-stimulated Tg level assessment and Dx-WBS at six months. Therapeutic responses were classified into four categories: Excellent Response (ER), Indeterminate Response (IDR), Biochemical Incomplete Response (BIR), and Structural Incomplete Response (SIR), with IDR, BIR, and SIR collectively termed as non-ER. ER was defined as negative imaging with suppressed Tg <0.2 ng/mL or TSH-stimulated Tg <1 ng/mL; BIR as negative imaging but suppressed Tg ≥1 ng/mL, stimulated Tg ≥10 ng/mL, or rising anti-Tg antibodies; SIR as any Tg level with structural or functional disease evidence, with or without anti-Tg antibodies; IDR as nonspecific imaging findings, faint thyroid bed uptake on RAI scanning, detectable non-stimulated Tg <1 ng/mL, stimulated Tg <10 ng/mL, or stable/declining anti-Tg antibodies without structural/functional disease. The therapeutic response assessment was conducted six months post-RAT, following the 2015 ATA guidelines (1). ER is defined as successful RRA.

### 2.3 Laboratory measurements

Serum TSH concentrations, sTg levels, and TgAb levels were measured by cobas e 801 analytical unit for immunoassay tests.

### 2.4 Post-therapy and diagnostic WBS

Rx-WBS was obtained 2–3 days after RRA using dualhead γ-cameras (Siemens Symbia T16 SPECT/CT) equipped with medium-energy collimators, set to a peak energy of 364 keV with a window width of 20%. Both anterior and posterior planar images, from the vertex to the knee, were acquired and stored in 256×1024 matrices using a scan speed of 5 to 10 cm/min. Dx-WBS was performed at 24–48 hours after oral administration of 74 to 185 MBq of ^131^I using the same camera and protocol as for the Rx-WBS described above. A nuclear medicine physician with 14 years of experience visually analyzed WBS images. Rx-WBS findings were classified as hot uptake in the thyroid bed of the neck or not. Diagnostic WBS was used to evaluate the treatment response of RRA by classifying remaining fainted uptake lesion in the neck or not.

### 2.5 Data analysis

Patient clinical information was acquired through electronic medical record system, and cancer stages were determined using the eighth edition of the American Joint Committee on Cancer (AJCC) staging system. Patients were categorized into Excellent Response (ER), Biochemical Incomplete Response (BIR), Structural Incomplete Response (SIR), and Indeterminate Response (IDR) groups based on six-month Dx-WBS findings and sTg levels. This retrospective study’s design received approval from the institutional review board of the Affiliated Hospital of Guilin Medical University, under approval number 19–000896. The requirement for informed consent was waived.

### 2.6 Statistical analysis

**Figure 1:**
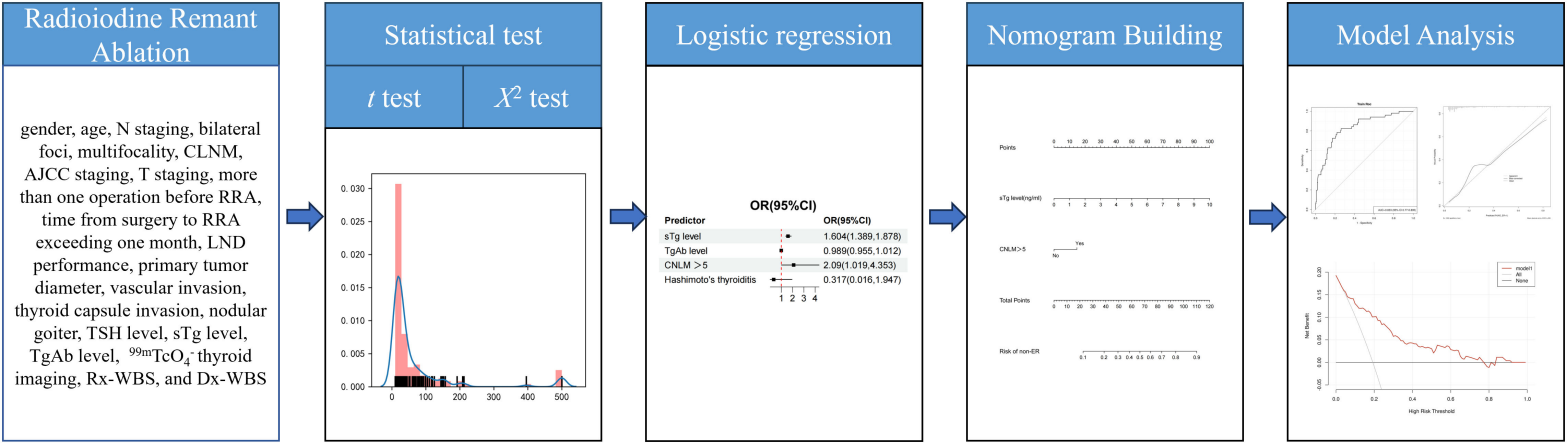
Workflow of this study. Univariate and multivariate logistic regression analyses were conducted to identify risk factors for intermediate-risk DTC post-RRA. A nomogram was developed from the result of multivariate logistic regression analyses. The nomogram’s performance was assessed using the concordance index (C-index) and evaluated through calibration curves and clinical decision curves, employing the Bootstrap method with 1,000 resamplings.

Statistical analyses were performed using R version 3.6.3 and Python version 3.7. In instances where baseline variables had missing values, multiple imputation analysis was conducted. The fully conditional specification discriminant function was utilized for categorical missing data, and the fully conditional specification regression was employed for continuous missing data. Continuous data are presented as means ± standard deviations (SD) for normally distributed data and medians with interquartile ranges (IQR) for non-normally distributed data. Categorical variables are expressed as frequencies and percentages. For comparisons, the chi-square test or Fisher’s exact test was used for categorical variables, and analysis of variance for continuous variables with a normal distribution. Univariate and multivariate logistic regression analyses were conducted to identify risk factors for intermediate-risk DTC post-RRA. Variables in univariate analysis included gender, age, N staging, bilateral foci, multifocality, CLNM, AJCC staging, T staging, more than one operation before RRA, time from surgery to RRA exceeding one month, LND performance, primary tumor diameter, vascular invasion, thyroid capsule invasion, nodular goiter, TSH level, sTg level, TgAb level, ^99m^TcO_4_^-^ thyroid imaging, Rx-WBS, and Dx-WBS. Multivariate analysis involved forward and backward selection procedures for parameters with P < 0.05 in log-rank tests, reporting odds ratios (ORs) with 95% confidence intervals (CIs). A nomogram was developed using the rms6.7.1 package in R version 3.6.3 (http://www.r-project.org/). The nomogram’s performance was assessed using the concordance index (C-index) and evaluated through calibration curves and clinical decision curves, employing the Bootstrap method with 1,000 resamplings. The workflow was shown in **Figure 1**.

## 3 Results

### 3.1 Clinicopathologic characteristics of patients

In this retrospective study, we analyzed 500 consecutive patients who underwent total thyroidectomy with CND and initial RRA treatment for intermediate-risk DTC at our institution between January 2018 and March 2023. From this cohort, we excluded 128 patients with sTg levels exceeding 10 ng/ml, 35 with serum thyroglobulin antibody (TgAb) levels above 115 U/mL, 52 with CLNM or DM identified Rx-WBS, 7 with CLNM or DM detected Dx-WBS at six months, 6 who were under 18 years old, and 7 lost to follow-up. Ultimately, 265 patients were included in the study. All patients were diagnosed with papillary thyroid carcinoma (PTC). The median age was 41 years (interquartile range: 34–50 years), with 28.7% (76/265) being male. The majority had T1 stage (83.4%), with more patients presenting with N1a disease (60.4%) than N1b; all were TNM stage M0, and 83.4% were AJCC stage I. Pre-RRA, 173 patients (65.3%) displayed hot uptake in the thyroid bed on ^99m^TcO_4_^-^ thyroid imaging, reducing to 0.4% (1 patient) post-RRA. Pre-RRA, 259 patients (97.7%) showed hot uptake in the thyroid bed on Rx-WBS, decreasing to 3.4% (9 patients) post-RRA as per Dx-WBS.

Significant differences were noted between the Excellent Response (ER) group and the non-ER group in terms of CLNM>5, Hashimoto’s thyroiditis, sTg level, Tg_off_ level, and TgAb level (P < 0.05). However, no statistically significant differences were found in gender, age, N staging, bilateral foci, multifocality, CLNM, AJCC staging, T staging, more than one operation before RRA, time from surgery to RRA exceeding one month, LND performance, primary tumor diameter, vascular invasion, thyroid capsule invasion, nodular goiter, TSH level, ^99m^TcO_4_^-^ thyroid imaging, Rx-WBS, and Dx-WBS between the two groups (P > 0.05). Detailed clinical characteristics of the patients are summarized in **Table 1**.

**Table 1:** Demographics and clinicopathologic characteristics of patients with intermediate-risk DTC after RRA.

Demographics or Characteristics	Item	General (n=265)	ER group (n=214)	Non-ER group (n=51)	*P*
Gender, n (%)	Male	76 (28.7)	60 (28.0)	16 (31.4)	0.636
	Female	189 (71.3)	154 (72.0)	35 (68.6)	
Age, median [IQR]		41.0 [34.0,50.0]	43.0 [34.0,50.0]	37.0 [33.0,46.0]	0.093
Primary tumor diameter, median [IQR]		1.2 [0.8,2.0]	1.2 [0.8,2.0]	1.2 [0.9,2.0]	0.858
AJCC stage, n (%)	I	254 (95.8)	205 (95.8)	49 (96.1)	0.927
	II	11 (4.2)	9 (4.2)	2 (3.9)	
T stage, n (%)	T1	221 (83.4)	177 (82.7)	44 (86.3)	0.539
	T2	44 (16.6)	37 (17.3)	7 (13.7)	
N stage, n (%)	N0	31 (11.7)	28 (13.1)	3 (5.9)	0.341
	N1a	160 (60.4)	128 (59.8)	32 (62.7)	
	N1b	74 (27.9)	58 (27.1)	16 (31.4)	
Vascular infiltration, n (%)	No	254 (95.8)	204 (95.3)	50 (98.0)	0.383
	Yes	11 (4.2)	10 (4.7)	1 (2.0)	
Thyroid capsule invasion, n (%)	No	179 (67.5)	140 (65.4)	39 (76.5)	0.130
	Yes	86 (32.5)	74 (34.6)	12 (23.5)	
Multifocality, n (%)	No	148 (55.8)	122 (57.0)	26 (51.0)	0.436
	Yes	117 (44.2)	92 (43.0)	25 (49.0)	
CLNM, n (%)	No	33 (12.5)	29 (13.6)	4 (7.8)	0.267
	Yes	232 (87.5)	185 (86.4)	47 (92.2)	
CLNM>5, n (%)	No	161 (60.8)	137 (64.0)	24 (47.1)	0.026
	Yes	104 (39.2)	77 (36.0)	27 (52.9)	
Time from surgery to RRA exceeding one month, n (%)	No	248 (93.6)	200 (93.5)	48 (94.1)	0.863
	Yes	17 (6.4)	14 (6.5)	3 (5.9)	
LND, n (%)	No	169 (63.8)	136 (63.6)	33 (64.7)	0.877
	Yes	96 (36.2)	78 (36.4)	18 (35.3)	
More than one operation before RRA, n (%)	No	260 (98.1)	211 (98.6)	49 (96.1)	0.235
	Yes	5 (1.9)	3 (1.4)	2 (3.9)	
Bilateral foci, n (%)	No	186 (70.2)	154 (72.0)	32 (62.7)	0.196
	Yes	79 (29.8)	60 (28.0)	19 (37.3)	
Hashimoto’s thyroiditis, n (%)	No	230 (86.8)	180 (84.1)	50 (98.0)	0.008
	Yes	35 (13.2)	34 (15.9)	1 (2.0)	
Nodular goiter, n (%)	No	167 (63.0)	137 (64.0)	30 (58.8)	0.490
	Yes	98 (37.0)	77 (36.0)	21 (41.2)	
^99m^TcO_4_^-^ thyroid imaging, n (%)	No thyroid remnant	92 (34.7)	73 (34.1)	19 (37.3)	0.672
	Thyroid remnant	173 (65.3)	141 (65.9)	32 (62.7)	
Rx-WBS, n (%)	No thyroid remnant	6 (2.3)	5 (2.3)	1 (2.0)	0.871
	Thyroid remnant	259 (97.7)	209 (97.7)	50 (98.0)	
Response to surgery, n (%)	ER	93 (35.1)	90 (42.1)	3 (5.9)	<0.001
	IDR	172 (64.9)	124 (57.9)	48 (94.1)	
Response to RRA, n (%)	BIR	2 (0.8)	0 (0.0)	2 (3.9)	0.999
	ER	214 (80.8)	214 (100.0)	0 (0.0)	
	IDR	49 (18.5)	0 (0.0)	49 (96.1)	
^99m^TcO_4_^-^ thyroid imaging after RRA, n (%)	No thyroid remnant	264 (99.6)	214 (100.0)	50 (98.0)	0.999
	Thyroid remnant	1 (0.4)	0 (0.0)	1 (2.0)	
Dx-WBS after RRA, n (%)	No thyroid remnant	256 (96.6)	214 (100.0)	42 (82.4)	0.999
	Thyroid remnant	9 (3.4)	0 (0.0)	9 (17.6)	
TSH Level, median [IQR]		86.0 [65.7,100.0]	85.2 [61.9,100.0]	88.7 [72.8,100.0]	0.508
TgAb level, median [IQR]		12.9 [10.0,21.2]	13.2 [10.0,24.1]	11.4 [10.0,15.1]	0.013
sTg level, median [IQR]		1.5 [0.5,3.5]	1.2 [0.4,2.8]	4.5 [2.6,7.3]	<0.001
Tg_off_ level, median [IQR]		0.1 [0.0,0.7]	0.1 [0.0,0.3]	2.4 [1.5,3.9]	<0.001

### 3.2 Development and validation of a non-ER predictive nomogram

**Figure 2:**
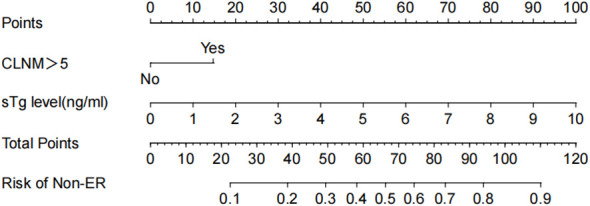
Nomogram for predicting non-ER risk. The value of each variable was scored on a point scale from 0 to 100, after which the scores for each variable were added together. That sum is located on the total points axis, which enables us to predict the probability of non-ER risk.

**Figure 3:**
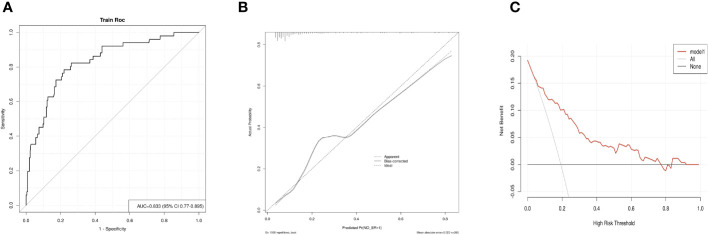
Evaluation of the nomogram model. **(A)** The Receiver Operating Characteristic (ROC) curve for the nomogram, derived using bootstrap resampling (1000 repetitions). **(B)** Calibration plot for the nomogram. The closer the performance nomogram’s solid line is to the dotted line of the ideal model, the higher the prediction accuracy of the nomogram. **(C)** Decision Curve Analysis (DCA) for the prediction model. The prediction model’s accuracy is represented by the red solid line, the gray line denotes the scenario where all patients exhibit non-complete response (non-ER), and the solid horizontal line implies that no patients experience non-ER. This graph illustrates the expected net benefit per patient in relation to the nomogram’s prediction of non-ER risk, with increased net benefit correlating to the extension of the model curve.

In this study, 215 patients (81.1%) exhibited an Excellent Response (ER) upon follow-up, while 48 (18.1%) demonstrated an Indeterminate Response (IDR), and 2 (0.8%) had a Biochemical Incomplete Response (BIR). Univariate analyses identified CLNM>5, Hashimoto’s thyroiditis, sTg level, and TgAb level as significant risk factors for a non-ER outcome following RRA. Factors such as gender, age, N staging, bilateral foci, multifocality, CLNM, AJCC staging, T staging, more than one operation before RRA, surgery to RRA duration exceeding one month, LND performance, primary tumor diameter, vascular invasion, thyroid capsule invasion, nodular goiter, TSH level, ^99m^TcO_4_^-^ thyroid imaging, Rx-WBS, and Dx-WBS were not significantly associated with non-ER outcomes. In multivariate analyses, CLNM>5 and sTg level emerged as independent risk factors for non-ER (**Table 2**). Logistic regression analysis was employed to estimate the likelihood of non-ER in patients with intermediate-risk DTC following RRA. In this model, specific point values were assigned to each predictor’s observed value (**Figure 2**), with the cumulative points for all variables representing an individual’s risk of non-ER post-RRA. The nomogram’s accuracy was rigorously validated using a bootstrap method with 1000 resamples, revealing an area under the curve (AUC) of 0.833 (95% CI = 0.770–0.895) (**Figure 3A**). The nomogram’s predicted probabilities were found to be in strong agreement with actual clinical outcomes (**Figure 3B**), and decision curve analysis demonstrated the model’s potential clinical applicability (**Figure 3C**).

**Table 2:** Multivariate analysis result (forward and backward selection procedures).

Predictor	Estimate	SE	Z	p	Odds Ratio	Lower	Upper
sTg level	0.502	0.075	6.714	0.0	1.652	1.436	1.928
CNLM>5	0.739	0.367	2.012	0.044	2.093	1.023	4.348
TgAb level	-0.011	0.014	-0.809	0.418	0.989	0.955	1.012
Hashimoto’s thyroiditis	-1.341	1.087	-1.234	0.217	0.262	0.014	1.48

### 3.3 Webserver development for the nomogram

For convenient application of our nomogram, we developed dynamic calculators on the basis of a user-friendly website (https://www.evidencio.com/models/show/10176), which could be used directly by researchers and clinicians. By inputting certain clinical variables, we can easily obtain the corresponding individualized predicted survival probabilities through the output data generated by the website.

## 4 Discussion

In this retrospective study, we found that sTg levels and CLNM>5 significantly contribute to the risk of non-ER in patients with intermediate-risk DTC following RRA. Utilizing these variables, we developed and validated a nomogram to estimate the risk of non-ER. This tool holds considerable potential importance for the primary prevention of non-ER in this patient population.

Contrary to previous research primarily centered on distinct factors influencing the prognosis of DTC post-surgery or radioactive iodine therapy, our study integrates clinicopathological characteristics and identifies that elevated sTg levels and CLNM>5 are associated with an increased risk of non-ER in patients with intermediate-risk DTC following RRA. Among these factors, elevated sTg levels were noted, corroborating findings from earlier studies (11, 16, 17). A retrospective analysis of 2,500 thyroid cancer patients established that a post-surgical thyroglobulin (ps-Tg) cutoff of ≤10.1 ng/mL predicts disease-free status with a negative predictive value of 95%. This threshold was consistently validated across all ATA risk categories. Additionally, the study revealed that a ps-Tg level of ≤10.1 ng/mL significantly reduces the likelihood of persistent or recurrent disease in patients classified as intermediate- and high-risk (18). In a prospective study of intermediate- to high-risk patients with sTg levels above 10 ng/mL, 28.4% displayed functional or structural disease following RAT. RAT, utilizing 5.55 GBq (150mCi) of Iodine-131, proved effective in detecting biochemical, functional, or structural disease, and elicited a substantial therapeutic response in a significant portion of these patients. Hence, these patients are deemed appropriate candidates for RAT (5). Therefore, in patients with a high risk of recurrence and sTg levels above 10 ng/ml, RAT has been shown to effectively enhance overall survival (OS) and disease-free survival (DFS). Consequently, it can be recommended as a standard treatment approach. Research on the risk factors of RRA in intermediate-risk DTC patients with low sTg levels (<10ng/ml) is relatively scarce. Previous studies examining the efficacy of total thyroidectomy and RRA in this demographic often included low-risk DTC patients, those with sTg levels over 10ng/ml, and patients with structural lesions in both Rx-WBS and Dx-WBS (19–22). Additionally, the variation in RRA dosages used in these studies contributes to biases in the research outcomes. This study specifically focuses on intermediate-risk DTC patients with sTg levels ≤10ng/ml and no structural lesions in either Rx-WBS or Dx-WBS. Furthermore, it standardizes the RRA dosage at 3.7GBq (100mCi) to enhance the reliability of the findings.

In patients with intermediate-risk DTC, current evidence regarding the impact of RRA on disease recurrence remains inconclusive, indicating beneficial effects in some cases but showing no benefit in others (23). The 2015 ATA guidelines advise that, following total thyroidectomy in patients with low-risk thyroid cancer or intermediate-risk disease exhibiting lower risk features (such as low-volume central neck nodal metastases without additional gross residual disease or adverse features), a lower administered activity of approximately 30 mCi for RRA is generally favored over higher administered activities (1). A retrospective study evaluated the therapeutic efficacy and long-term clinical outcomes of varying doses of RRA in patients with intermediate-risk DTC. This study involved 204 intermediate-risk DTC patients, with 124 receiving a high dose (3.7 or 5.55 GBq) and 80 receiving a low dose (1.11 GBq) of radioactive iodine. The findings indicated no significant difference in treatment success rates between the high-dose (54.84%) and low-dose (45.00%) groups, regardless of whole-body scan results. According to the American Thyroid Association’s reclassification system, the post-treatment response rates were as follows: ER (high-dose 54.84%, low-dose 45.00%), IDR (high-dose 34.68%, low-dose 30.00%), BIR (high-dose 4.03%, low-dose 13.75%), and SIR (high-dose 6.45%, low-dose 11.25%). Additionally, long-term follow-up showed recurrence rates of 5.65% in the high-dose group and 8.75% in the low-dose group. Consequently, the study suggests that in high-iodine intake regions of Korea, low-dose radioactive iodine therapy may be less effective for treating intermediate-risk DTC patients, potentially necessitating additional treatment in the low-dose group (19). A retrospective study evaluated the efficacy of low-dose (1110 MBq) versus high-dose (2960–3700 MBq) RRA in patients with intermediate-to-high-risk DTC. This study found no significant difference in initial success rates between the low-dose (73.5%) and high-dose (70.6%) groups, though high-dose RRA may be more effective for high-risk patients (20). Another study concluded that low-dose ^131^I is as effective as high-dose in RRA of papillary thyroid carcinoma patients. In the intermediate-risk group, disease-free survival rates at 6 months, 1 year, and 2 years were marginally higher in the high-dose group compared to the low-dose group (24). Collectively, these studies indicate that the selection of RRA dosage for intermediate-risk DTC patients should be individualized, considering factors such as disease risk and pre-treatment thyroglobulin levels, instead of implementing a standardized dosage approach. The efficacy of low-dose RAI therapy, especially in specific patient subsets, underscores the importance of meticulous patient selection and monitoring. Consequently, we performed a retrospective analysis of risk factors in patients with intermediate-risk DTC who underwent 3.7 GBq (100 mCi) RRA therapy at our center. Our evaluation suggests that these patients may face an elevated risk of recurrence and higher sTg level. We also successfully developed a predictive nomogram for intermediate-risk DTC patients undergoing RRA dosage at 3.7GBq (100mCi). Furthermore, a web-based dynamics calculator was created for use by clinicians at our center and other institutions.

The 2015 ATA guidelines introduced revised risk stratifications compared to the 2009 version (1). Under this framework, high risk is characterized by lymph node metastases larger than 3 centimeters, while intermediate risk involves either palpable disease or more than five lymph nodes (CLNM > 5), each smaller than 3 centimeters. Yun et al. (25) determined that the optimal cutoff value for lymph node metastases (LNMs) impacting RAI treatment response is five. For patients with CLNM > 5, the most effective lymph node ratio (LNR) cutoff was identified as 0.30. Factors such as CLNM > 5, gender, lymph node dissection, and ATA risk classification were found to be independent predictors of RAI response. Correspondingly, another study indicated that patients with over five metastatic lymph nodes exhibit more aggressive clinicopathological features and poorer outcomes (26). Echoing our study’s results, these findings underscore the importance of both the quantity and characteristics of lymph node metastases in determining thyroid cancer prognosis. They further stress the need for precise assessment and monitoring of lymph node involvement to inform effective treatment plans and predict patient outcomes.

Recent research has advanced the development of nomograms, offering highly accurate prognostic information for thyroid cancer, surpassing traditional methods like the AJCC staging system (27–29). A study based on SEER data crafted a nomogram to predict the risk of DM and its prognostic value in female patients with DTC (30). Additionally, a nomogram has been created to forecast the cancer-specific survival (CSS) of patients with poorly differentiated thyroid carcinoma, aiding clinicians in formulating suitable treatment strategies (31). Further, specialized survival nomograms for patients with DTC and DM have been developed, enhancing prognostic understanding in this subgroup (32). Collectively, these studies represent significant advancements in predictive modeling for thyroid cancer, offering more precise and customized prognostic tools that could markedly influence patient management and treatment approaches. We aspire for our nomogram to be utilized in clinical practice, providing personalized predictions of RRA efficacy for intermediate-risk DTC patients, and facilitating individualized treatment options. We also eagerly anticipate collaborating with researchers from other centers to refine this clinical prediction model further.

This study is subject to several limitations: (1) As a retrospective analysis, it may be affected by recall bias and incomplete data, necessitating further prospective studies for confirmation of results. (2) Being a single-center study with a relatively small sample size, its findings may lack generalizability, underscoring the need for multi-center studies to verify these results. (3) The study primarily focuses on certain clinical and pathological markers as predictors, which might neglect other vital factors affecting treatment outcomes. Notably, the BRAF^V600E^ mutation, demonstrated by preclinical studies to significantly diminish sodium-iodide symporter expression and reduce RAI uptake, along with its correlation with ER rates and AXL expression in DTC patients, could influence results. Regrettably, only a limited subset of our cohort underwent testing for the BRAF^V600E^ mutation. Future research models should incorporate genomic data to improve prognostic accuracy for responses to RRA across a more diverse patient population. (4) The outcome selected—response to RRA—may not accurately reflect the recurrence risk and overall survival of DTC patients. Despite this, the 2015 ATA guidelines indicate that DTC patients categorized as intermediate or high risk could significantly reduce their risk of recurrent or persistent disease by achieving an ER to RRA. Additionally, in a previous study with a protracted follow-up period, no patients transitioned from an ER outcome to a non-ER outcome six months post-treatment (33). These observations highlight the utility of RRA response as an effective and practical indicator for predicting clinical outcomes concerning both recurrence and specific mortality risks, particularly in DTC, which is typically slow-growing. However, a longer follow-up period for the cohorts in this study is necessary to verify the predictive capacity of this nomogram for DFS and overall survival in DTC patients after RRA, and to identify variations in prognostic factors across different outcomes. (5) Although a nomogram and an online calculator were developed, external and clinical validations are essential before their practical implementation.

## 5 Conclusion

This study reveals that in intermediate-risk DTC patients, the likelihood of an non-ER to RRA is significantly linked with the presence of CLNM>5 and higher sTg levels. A predictive model incorporating these factors has been established to assess the risk of Non-ER, accompanied by the development of a network-based dynamic calculator. This model has shown high accuracy in validation, aligning closely with actual clinical outcomes. This tool not only assists in treatment decision-making but also enhances the precision of prognosis information. Nevertheless, additional research and validation are required.

## Data availability statement

The original contributions presented in the study are included in the article/supplementary material. Further inquiries can be directed to the corresponding authors.

## Ethics statement

The studies involving humans were approved by the ethics committee of the Affiliated Hospital of Guilin Medical University. The studies were conducted in accordance with the local legislation and institutional requirements. Written informed consent for participation was not required from the participants or the participants’ legal guardians/next of kin in accordance with the national legislation and institutional requirements.

## Author contributions

LL: Data curation, Methodology, Writing – original draft. QL: Data curation, Writing – review & editing. ZG: Formal analysis, Writing – review & editing. YL: Formal analysis, Supervision, Validation, Writing – review & editing. CL: Data curation, Writing – review & editing. JL: Data curation, Writing – review & editing. JH: Data curation, Writing – review & editing. XM: Writing – review & editing. WF: Writing – review & editing.
